# Assessment of fighting ability in the vocal cichlid *Metriaclima zebra* in face of incongruent audiovisual information

**DOI:** 10.1242/bio.043356

**Published:** 2019-12-18

**Authors:** M. Clara P. Amorim, Paulo J. Fonseca, Nicolas Mathevon, Marilyn Beauchaud

**Affiliations:** 1 MARE–Marine and Environmental Sciences Centre, ISPA-Instituto Universitário, 1149-041 Lisbon, Portugal; 2 Departamento de Biologia Animal and cE3c - Centre for Ecology, Evolution and Environmental Changes, Faculdade de Ciências, Universidade de Lisboa, 1749-016 Lisbon, Portugal; 3 Equipe de Neuro-Ethologie Sensorielle ENES/CRNL, University of Lyon/Saint-Etienne, CNRS UMR5292, INSERM UMR_S 1028, 42023 Saint-Etienne, France

**Keywords:** Incongruent signals, Multimodal communication, Opponent assessment, Adaptive framework, Receiver psychology, Cichlids

## Abstract

Information transfer between individuals typically depends on multiple sensory channels. Yet, how multi-sensory inputs shape adaptive behavioural decisions remains largely unexplored. We tested the relative importance of audio and visual sensory modalities in opponent size assessment in the vocal cichlid fish, *Metriaclima zebra*, by playing back mismatched agonistic sounds mimicking larger or smaller opponents during fights of size-matched males. Trials consisted in three 5-min periods: PRE (visual), PBK (acoustic+visual) and POST (visual). During PBK agonistic sounds of smaller (high frequency or low amplitude) or larger (low frequency or high amplitude) males were played back interactively. As a control, we used white noise and silence. We show that sound frequency but not amplitude affects aggression, indicating that spectral cues reliably signal fighting ability. In addition, males reacted to the contrasting audio-visual information by giving prevalence to the sensory channel signalling a larger opponent. Our results suggest that fish can compare the relevance of information provided by different sensory inputs to make behavioural decisions during fights, which ultimately contributes to their individual fitness. These findings have implications for our understanding of the role of multi-sensory inputs in shaping behavioural output during conflicts in vertebrates.

## Abstract (precis)

**Summary:** Cichlid fish rely on the sensory channel indicating higher risk when facing incongruent visual and acoustic information during opponent assessment.

## INTRODUCTION

Multimodal communication, i.e. the use of distinct signalling channels to transfer related information, can be a powerful means to reinforce the efficiency of information transfer, since different modalities can provide independent measurements of an event (Narins et al., 2003; Partan and Marler, 2005; Munoz and Blumstein, 2012; Fetsch et al., 2013). However, sensory modalities are associated with some degree of uncertainty (associated with signal-to-noise ratio) which can lead to perception errors and costly decision-making (Munoz and Blumstein, 2012). In addition, as noise in one modality may be unrelated to the noise in another, sensory incongruity may arise when noise affects mainly one modality (De Gelder and Bertelson, 2003). Multisensory-based decisions taking into account uncertainty and congruency of sensory cues is thus a challenge that animals, including humans, must face to improve behavioural decisions (Ernst and Banks, 2002; Fetsch et al., 2013; McIntyre and Preuss, 2019).

Recent years have witnessed a growing interest concerning multimodal perception and communication (Partan and Marler, 2005; Munoz and Blumstein, 2012; Smith and Evans, 2013), both in terms of neurophysiological and psychological approaches, and mostly focusing on how perceptive mechanisms deal with uncertainty reduction (Fetsch et al., 2013; Schumacher et al., 2017). For example, during speech perception humans rely more on the visual modality when noise increases in the acoustic modality and auditory illusions may even arise when lip movements do not match auditory cues (the McGurk effect; McGurk and MacDonald, 1976). As another example, Schumacher et al. (2016) showed that individuals from the weakly electric fish, *Gnathonemus petersii*, weight object-related visual and electric sensory inputs according to their reliability to minimize uncertainty during object recognition.

However, little is known on how multi-sensory inputs are integrated to produce adaptive behavioural decisions (Kayser et al., 2010; Munoz and Blumstein, 2012; Taylor and Ryan, 2013; Ben-Ari and Inbar, 2014; Kozak and Uetz, 2016). This is particularly important in a high-risk context, such as when facing a predator or a dangerous opponent, as the costs of taking wrong decisions may be critically high. For example, to make an adequate decision, pea aphids dynamically link an unreliable cue, e.g. air movement or plant vibrations, which can be due to either wind or the proximity of a mammalian predator, with a reliable cue from another sensory source, such as hot and humid air deriving from the mammal's breath (Ben-Ari and Inbar, 2014). Here, different interpretations of the unreliable cue can lead to opposing reactions: avoid dislodgement by holding on to the host plant or drop to the ground to avoid predation but facing other risks. Ben-Ari and Inbar (2014) have shown that these animals use the time lag between unreliable cues and a reliable cue for mammalian presence to finely modulate their response and thus avoid costly wrong decisions.

Here we investigate how a territorial fish deals with concurrent information about a competitor's body size transferred by acoustic and visual channels using an incongruent signal paradigm. Body size is a key feature in the biology of animals (Andersson, 1994), including humans (Smith et al., 2005; Klofstad et al., 2012), as it often represents an important determinant of social status, fighting ability and reproductive success (Davies and Halliday, 1978; Andersson, 1994; Arnott and Elwood, 2009). Many animals assess their congeners' size through direct visual cues or relying on indirect vocal displays (Bradbury and Vehrencamp, 2011). Honest information on body size conveyed by sounds can be mediated through their spectral properties as larger vocal organs and vocal tracts produce and radiate lower frequencies more efficiently (Bradbury and Vehrencamp, 2011). This is the case of formant spacing in mammals (Reby and McComb, 2003), including humans (Pisanski et al., 2014), fundamental frequency in anurans (Davies and Halliday, 1978) or dominant frequency in fish (Myrberg et al., 1993; Bertucci et al., 2012). Sound amplitude can also reflect body size since larger animals may produce louder calls due to scaling effects (Gerhardt, 1975; Amorim et al., 2013). However, both spectral and amplitude cues may be modulated by the sender, e.g. according to social context (Ritschard et al., 2012) and background noise (Brumm and Slabbekoorn, 2005), and the reliability of sound-mediated body size information is sometimes questionable (Bee et al., 2000). How animals deal with conflicting information brought by the visual channel and the acoustic channel remains an open question.

Teleost fishes provide excellent model systems to investigate the multisensory audio-visual-based decisions during mutual assessment in vertebrates, since there is a remarkable conservation of perception and social decision-making networks across vertebrates (O'Connell and Hofmann, 2011). In addition, many fish species produce simple stereotyped agonistic sounds with size-dependent spectral and amplitude features (Ladich and Myrberg, 2006). In contrast with anurans, birds and mammals (Bradbury and Vehrencamp, 2011), most fishes are not known to adjust these acoustic features (for exceptions see Amorim and Almada, 2005; Holt and Johnston, 2014), offering simpler models to investigate.

**Fig. 1.**
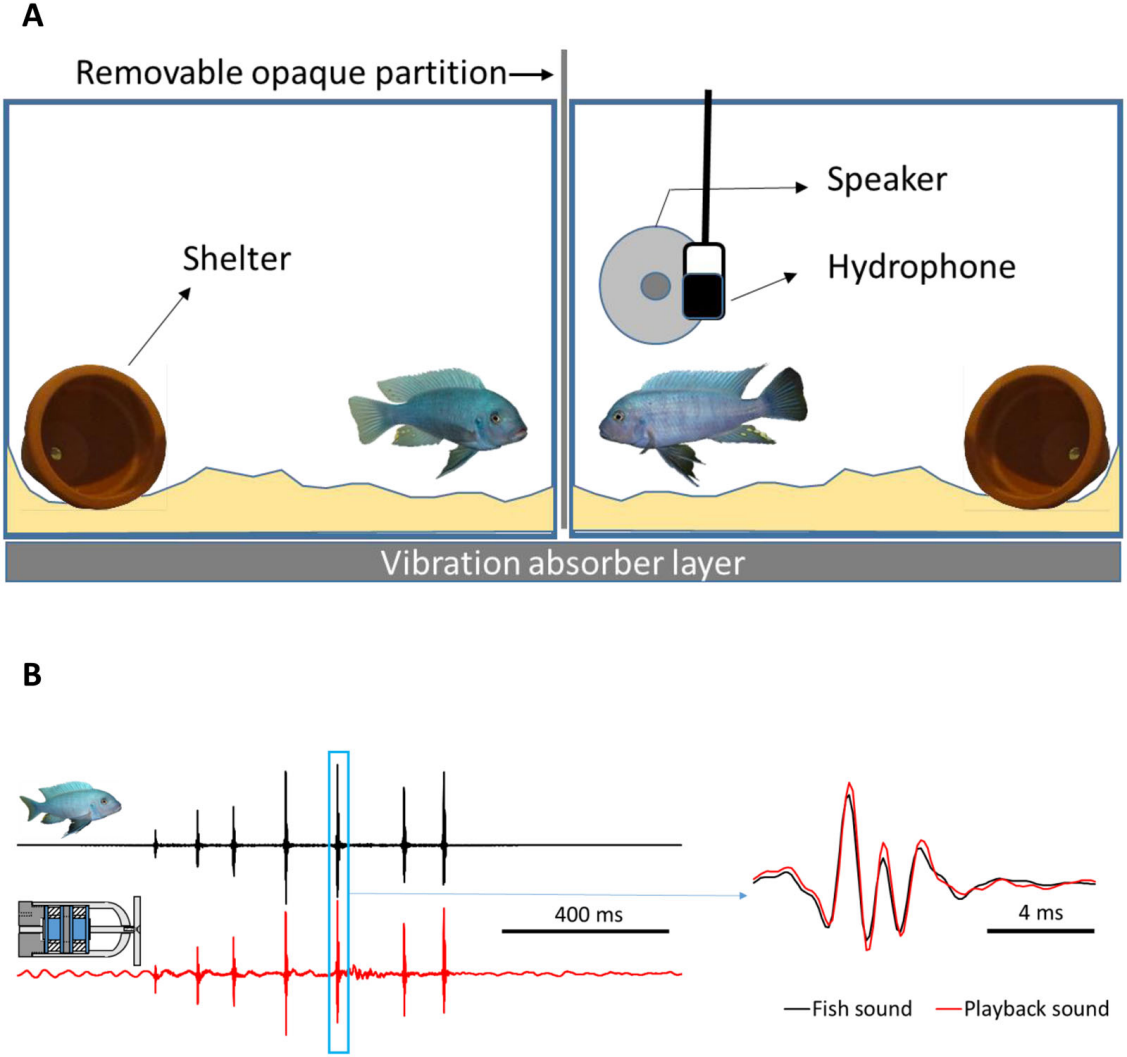
**Experimental playback setup.** (A) Unfamiliar size-matched *Metriaclima zebra* males were placed one in each aquarium 1 day before trials. Trials started by removing the opaque partition to allow agonistic interactions and consisted in three 5 min periods: pre-playback (PRE), acoustic stimuli presentation (PBK) and post-playback (POST). During the PBK period, 10 acoustic stimuli (agonistic sounds or control stimuli) were played back when the opponent interacted agonistically with the subject. (B) Oscillograms of an agonistic sound (left) produced by a male (black) or played back by the speaker (red) are depicted. The detail of one sound pulse is shown on the right.

The African cichlid *Metriaclima zebra* (Boulenger 1899) is a vocal territorial fish for which body size is a critical determinant of fight outcome (Simões et al., 2008; Mellor et al., 2012). Males of this species use visual and acoustic cues (Simões et al., 2008; Bertucci et al., 2012), and likely chemical and vibrational (lateral line) cues, in male–male fighting ability assessment and in a reproductive context (Butler and Maruska, 2015; Escobar-Camacho and Carleton, 2015; Keller-Costa et al., 2016). During agonistic interactions, *M. zebr*a male competitors perform conspicuous visual lateral displays while producing pulsed sounds (Fig. 1) with size-dependent acoustic features [larger individuals emitting lower pitched and louder acoustic signals (Simões et al., 2008; Bertucci et al., 2012)]. *Metriaclima zebra* males show increased aggressiveness in response to the sight of a real contestant but no response to dominant male urine or agonistic sounds presented separately (Chabrolles et al., 2017). However, when agonistic sounds are combined with visual information, aggressiveness decreases (Bertucci et al., 2010) making this an ideal model system to assess which sensory channel becomes prevalent in shaping behavioural decisions during an interaction with a competitor.

In the present study, we experimentally tested how fish deal with incongruent acoustic and visual information, i.e. when the frequency or the amplitude of the emitted sounds do not match the actual body size of the emitter. Given the potential costs of fight outcome, we hypothesized that the sensory channel (acoustic or visual) that prevails in modulating agonistic interactions is the one conveying the higher threat information. Fish challenged with playback of agonistic sounds in the presence of a visible opponent should thus decrease aggression levels in response to the sensory channel, and signal component (sound frequency versus amplitude), indicating a larger opponent.

## RESULTS

**Fig. 2.**
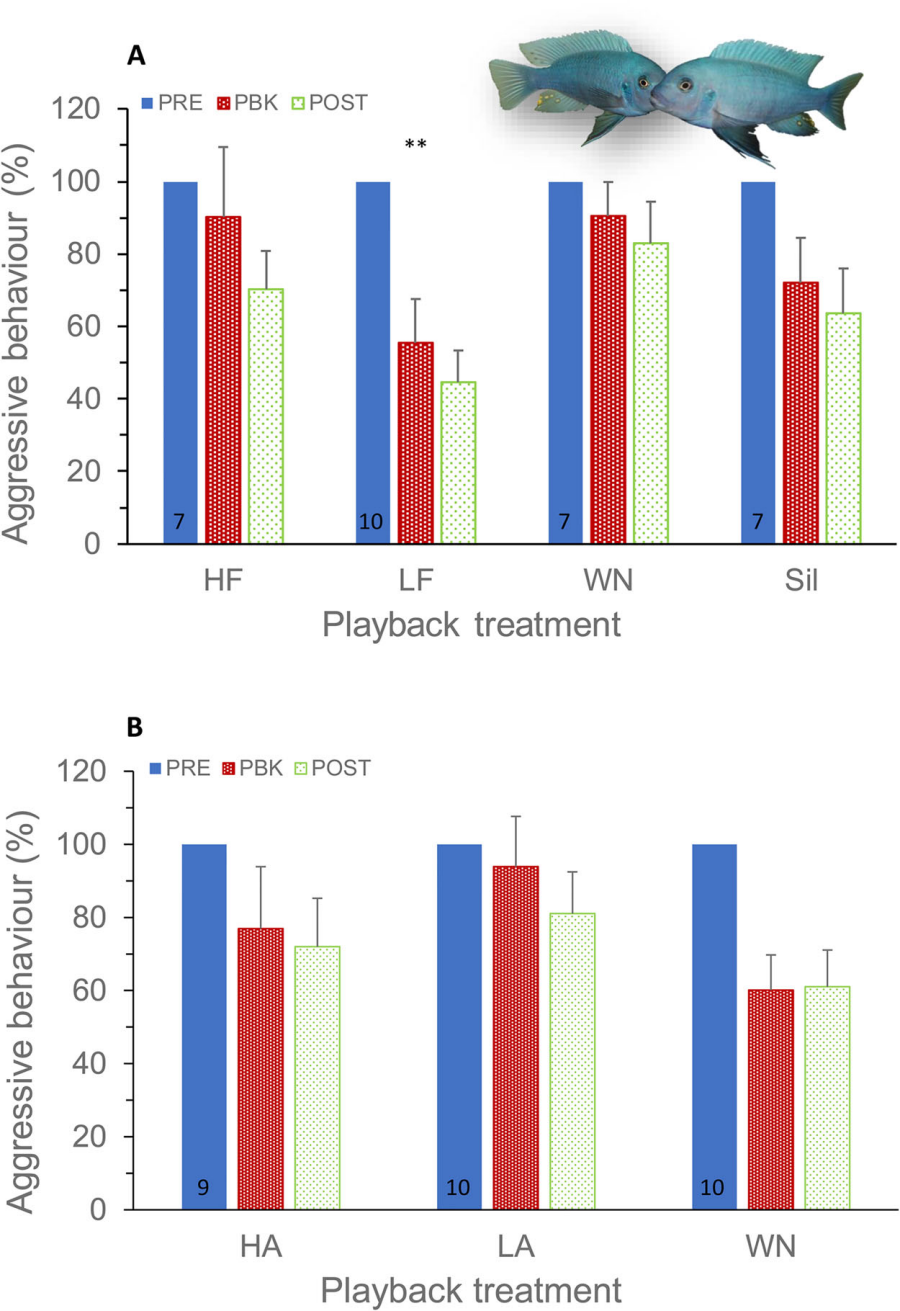
**Aggressive reaction of male fish to incongruent audio and visual opponent cues.** (A) Test fish exposed to agonistic sounds with modified spectral cues. (B) Test fish exposed to agonistic sounds with modified amplitude cues. The decrease in aggression level along trial periods was significantly steeper in the LF group than in the SIL group. Mean±s.e.m. of raw data are shown expressed as a percentage of the baseline; sample size per group is presented in the PRE column; **P<0.01 determined by a linear mixed model.

In experiment 1, the opponent male was matched in size to the tested individual, but the spectral content of the playback came from either a smaller (high frequency; HF) or a larger (low frequency; LF) male (Fig. 1). Playback treatment had a significant effect on the subject's aggression level (*F*_3,64.3_=6.02, *P*=0.001; Tables 1 and 2; Table S1). Although there was a general decrease of total agonistic behaviour with trial period (*F*_1,73.4_=4.62, *P*=0.04), the decrease was more marked when fish were exposed to low-frequency sounds than to the remaining treatments (trial period×treatment, *F*_3,57.6_=4.55, *P*=0.006; Fig. 2A). The decrease in aggression level along trial periods was significantly steeper (*t*=−2.75, *P*=0.008) in the LF group than in the silent group; the slopes for HF and white noise (WN) were not significantly different (*P*>0.05) from the silent groups (baseline) (Table 1). Under the LF treatment, subject males reduced the total number of agonistic behaviours by −1.79 to −11.39 in relation to silent groups (95% confidence intervals for the LF×period in Table 1). Agonistic behaviour of the opponent had a significant effect on the subject's aggressive behaviour (*F*_1,80.8_=16.92, *P*<0.001).

**Table 1.**
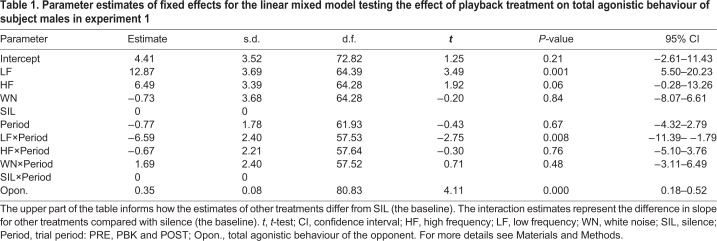
**Parameter estimates of fixed effects for the linear mixed model testing the effect of playback treatment on total agonistic behaviour of subject males in experiment 1**

**Table 2.**
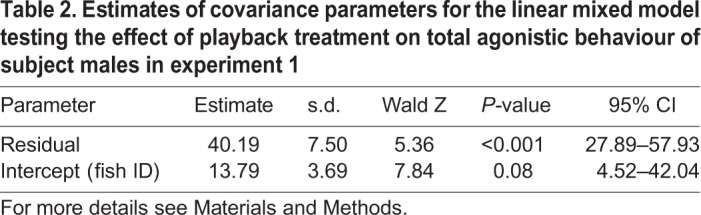
**Estimates of covariance parameters for the linear mixed model testing the effect of playback treatment on total agonistic behaviour of subject males in experiment 1**

In experiment 2, the opponent male was similarly visually matched in size to the tested individual, but the amplitude of the played back sounds was either increased (high amplitude; HA) or decreased (low amplitude; LA), mimicking sounds produced by a larger or smaller male. In contrast with experiment 1, agonistic behaviour was not affected by playback treatment (playback treatment, *F*_2,38.3_=0.85, *P*=0.44). As in experiment 1, there was a decrease of total agonistic behaviour with trial period (*F*_1,57.2_=7.30, *P*=0.009), but no significant interaction between playback treatment and trial period (*F*_2,54.9_=1.02, *P*=0.37; Fig. 2B; Table S1), suggesting that agonistic sound amplitude does not modulate fighting interactions. Agonistic behaviour of the opponent similarly had a significant effect on the subject's aggressive behaviour (*F*_1,80.0_=24.99, *P*<0.001).

## DISCUSSION

Our experiments suggest that fighting *M. zebra* males rely on the sensory channel conveying the most threatening information to assess an opponent. The audio and visual sensory channels thus seem to have variable weights in decision-making depending on which channel signals the larger male. Moreover, the frequency spectrum of agonistic sounds, but not their amplitude, affected the aggressive response of fish to a visible competitor. Spectral cues may thus be more reliable than amplitude cues to signal fighting ability.

We observed a decrease in aggression level along trial periods in all treatments likely because fish did not have direct physical contact preventing fight escalation. However, the decrease in aggression level along trial periods was steeper in the low-frequency sound playback group than in the remaining groups. These results indicate that the playback of low-frequency sounds, mimicking a larger male, significantly decreased the subject's level of aggression while the high frequency sounds of smaller males and the control stimuli had no effect. The decrease in aggression in response to a threat during exposure to sounds from similar sized males is consistent with previous experimental results in *M. zebra* (Bertucci et al., 2010) and other fish species (Stout, 1963; Schwarz, 1974; Rigley and Muir, 1979; Ladich et al., 1992).

**Fig. 3.**
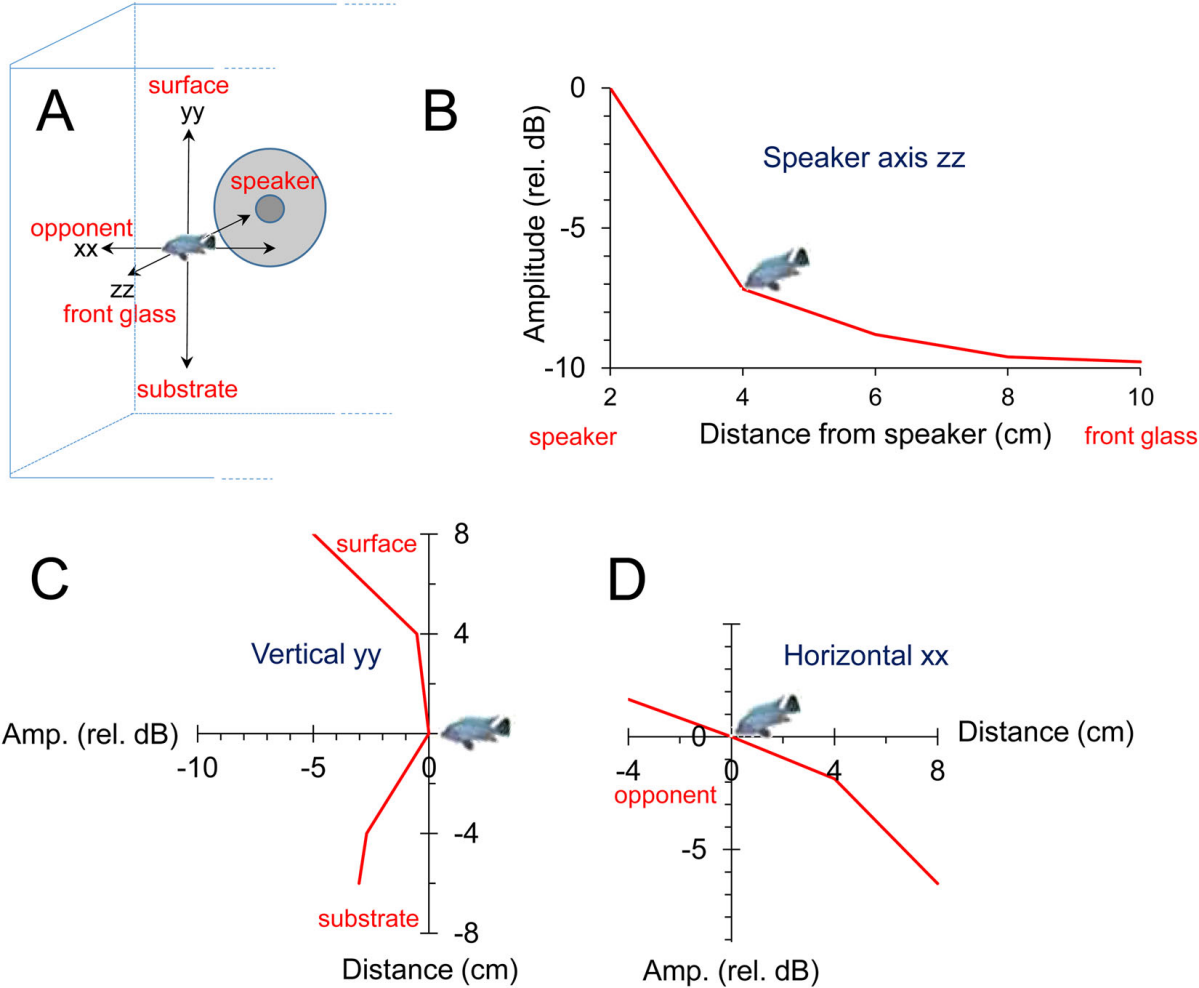
**Variation of the average RMS amplitude of played-back fish sounds measured in several positions in front of the speaker along three axes.** (A) Attenuation in the region of the aquarium where agonistic interactions occurred. (B) Attenuation along the speaker axis (zz – from the speaker to the front glass). Values are represented relative to the measurement obtained with the hydrophone 2 cm away from the speaker. (C,D) Variation of the sound field in a plane 4 cm in front of the speaker. 0 (depicted by the fish icon) corresponds to the measurement at 4 cm in A and represents a position typically held by the subject fish.

**Fig. 4.**
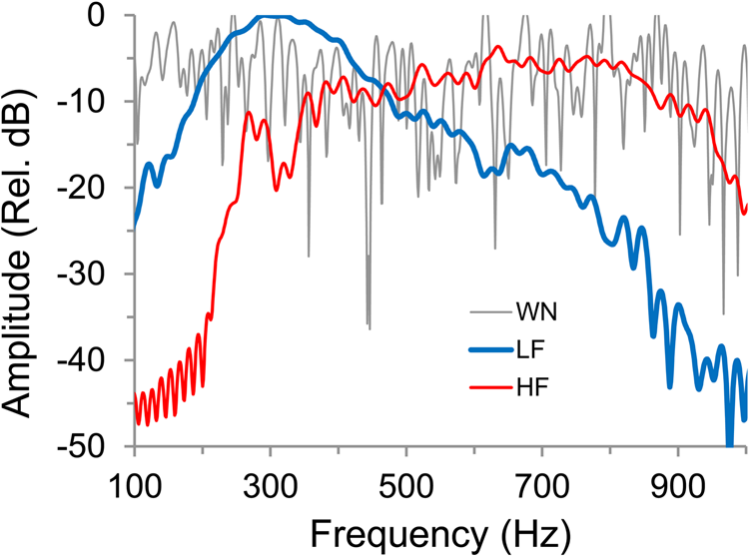
**Examples of spectra of LF, HF and WN acoustic stimuli.** The LF sound has an instantaneous frequency of 291 Hz and the HF of 635 Hz and were produced by males with a SL of 10. 6 cm and 6.2 cm, respectively. Spectra were calculated with a 1024 points FFT, Hanning window.

Conversely, we have found no effect of amplitude variation on aggressiveness. This result could be due to sound amplitude being highly dependent on propagation distance, especially close to the source (typical distances of interacting fish), in contrast with the spectral components of acoustic signals that are less distance-dependent (Mann, 2006; Alves et al., 2016) (Figs 3 and 4). Frequency cues thus appear to be a more reliable indicator of the sender's size than sound amplitude. In addition, in fish (Parmentier and Fine, 2016) and across taxa (Bradbury and Vehrencamp, 2011), there is a well-established relation between sound spectral properties and body size. In cichlids the structures involved in sound production and radiation are not yet known but there is a marked and tight relationship between dominant frequency and fish size (Bertucci et al., 2012; Amorim and Almada, 2005). This relation is likely derived from scaling of muscles and associated structures involved in sound production (Parmentier and Fine, 2016). The relation between sound amplitude and body size in fish, including cichlids, is less studied, but it can also be mediated by scaling effects; e.g. larger swimbladders amplify and radiate sound more efficiently (Parmentier and Fine, 2016). The importance of sound level in male–male assessment in fish has rarely been addressed but should not be excluded for other fish species. For example, Ladich (1998) showed that, in parallel to body size*,* lower dominant frequency and higher sound pressure levels of agonistic sounds (features correlated with larger body size) were good predictors of winning a fight in male croaking gouramis, *Trichopsis vittata*. However, this study did not disentangle the role of spectral and amplitude sound properties as the present study.

Consistent with our results, research in psychophysics and mathematical psychology has established that humans and other animals reduce perceptual uncertainty and optimize behavioural choices by weighing the reliability of the different available cues (Partan et al., 2010; Trommershäuser et al., 2011; Fetsch et al., 2013; Seilheimer et al., 2014; Kayser and Shams, 2015). Recently, Schumacher and colleagues (Schumacher et al., 2016, 2017) have shown that the weakly electric fish (*G.* *petersii*), when presented with an object recognition task involving sensory conflict, also weight sensory inputs dynamically according to their reliability to minimize uncertainty and to optimize sensory integration. The results from the present study are in line with these studies and suggest that cichlid fish gave prevalence to either the acoustic or the visual channel depending on the imparted information indicating that, as weakly electric fish, cichlids may be capable of dynamic weighting of different sensory inputs in a social interaction. Future work, where different fish sizes (large and small) are combined with high- and low-frequency sounds, should be carried out to ascertain this possibility.

Although multimodal communication and perception has received increasing attention in recent years (Narins et al., 2003; Skals et al., 2005; Koppen and Spence, 2007; Diaconescu et al., 2011; Pluta et al., 2011; Taylor et al., 2011; Fetsch et al., 2013; Ben-Ari and Inbar, 2014; Cecere et al., 2015; Kayser and Shams, 2015; Parise and Ernst, 2016; Schumacher et al., 2016, 2017; Halfwerk et al., 2019; McIntyre and Preuss, 2019; Mitoyen et al., 2019), multisensory perception should not only be interpreted in relation to information uncertainty but also in an adaptive framework, i.e. taking into account the differential costs in decision-making under different cost–benefit scenarios (Munoz and Blumstein, 2012).

In addition, to better understand animal communication, responses to multimodal signals and their unimodal components should be compared. Considering a receiver's psychology framework (Partan and Marler, 2005), which has provided a foundation to study multimodal communication, acoustic and visual cues could be considered non-redundant in *M. zebra*, as they elicit different behavioural responses when presented alone (acoustic signals elicit no reaction, while visual cues increase aggression; Bertucci et al., 2010; Chabrolles et al., 2017). The composite signal, however, could be classified differently depending on the ecological scenario as it evokes different responses. When visual cues are combined with sounds from a smaller fish (high frequency) or with sounds encoding no reliable size information (matched-size frequency with different amplitudes), the response remains unchanged (dominance), but when associated with sounds from a larger opponent (low frequency) aggressiveness decreases (modulation). This model system thus affords the opportunity for future studies to assess the relative contribution of each modality on behavioural outcome under different ecologically relevant cost–benefit conditions.

In conclusion, our work suggests that cichlid fish have the ability to assess signal reliability but also to weight the information provided by different sensory inputs when making behavioural decisions that affect fight outcome. The present findings contribute to the understanding of the evolution of multimodal signals in an adaptive framework.

## MATERIALS AND METHODS

### Fish

Experiments were performed with fish purchased from a local supplier (Kingersheim, France) 1 month before the experiments. The animals were kept in two stock tanks (120 cm×60 cm×50 cm) at the Equipe de Neuro-Ethologie Sensorielle (ENES) laboratory. Each tank held 15 adult males that were fed daily with commercial cichlid food (JBL NovoMalawi sticks for Malawi cichlids, JBL NovoRift, and cichlid flakes, Tetra). PVC tubes and bricks were provided as shelters. The tanks were maintained at 25±1°C with a pH of 8.0 and a 12:12 h light–dark cycle.

### Experimental design

The study consisted of two different playback experiments aimed at testing separately the relevance of the two main acoustic features that code for a competitor's size: sound frequency spectrum (experiment 1) and sound amplitude (experiment 2). Bertucci et al. (2012) showed that sound pulse instantaneous frequency varies from 500–650 Hz to c. 300 Hz with a 4 cm increase in male standard length (SL). As there are no reports on the relation between sound amplitude and male size in this species, we measured the relative peak amplitude from sounds of three vocal males (1–8 sounds per male; SL of individuals ranged between 8.4 cm and 9.3 cm). Peak amplitude was measured at the spectral peak of sounds recorded from fish at c. 4 cm from the hydrophone (see below). We observed that in these three males, peak amplitude increased c. 11 dB with a 0.9 cm increase in SL, i.e. c. 3.5-fold in linear scale. If *M. zebra* males use spectral information and/or sound amplitude to assess an opponent's size, they are expected to escalate fights less with an opponent producing lower frequency and/or louder sounds characteristic of larger fish in comparison with males perceived as smaller. Note that agonistic interactions occurred often between males of different sizes in stock tanks in our facilities, so aggression is expected to occur even when there are perceived asymmetries in the opponent's size, with differences in body size determining aggression level and fight outcome (Mellor et al., 2012). In addition, as fish were matched in size, but the delivered acoustic signals provided mismatched size-information, the experiments aimed to test the prevalence of the acoustic versus the visual sensory channels in fish fights.

### Playback setup

Two experimental aquaria (60 cm×30 cm×30 cm) separated by a removable opaque partition were placed on a shelf insulated from floor vibrations inside a sound-attenuating chamber (Fig. 1A). To reduce tank resonance and reflections, aquaria walls were lined on the inside with air-bubble packing film (following Wysocki and Ladich, 2002) except for the front and the opponent side panels.

The floor of each aquarium was covered with a 3–4 cm layer of sand substrate and contained a terracotta pot (shelter). A filter, aerator and internal heater were positioned opposite to the subject–opponent interaction area and surrounded by a plastic net. The test (right) aquarium also contained a custom-made underwater loudspeaker placed in the subject–opponent interaction area and protected by a plastic net to exclude fish from the speaker area. Note that because of its size, the speaker was placed laterally to the visual stimulus (opponent) thus creating a spatial disparity between the visual and acoustic signals (Fig. 1A). Although this disparity may have influenced behavioural responses due to playback, a similar experimental setup has been shown to elicit behavioural changes in this species (Bertucci et al., 2010). We positioned a hydrophone (Aquarian Audio Products H2a-XLR, AFAB Enterprises, Anacortes, WA, USA; sensitivity: −180 dB re 1 V µPa^−1^, flat frequency response±4 dB 20 Hz–4.5 kHz) 2 cm in front of the speaker to record playback stimuli and sounds produced by subject fish.

The playback chain consisted of the custom-made underwater speaker and a driver (Fonseca and Maia Alves, 2012), which are able to reproduce low-frequency pulsed fish sounds with great accuracy (Fig. 1B). Sound stimuli were fed to the driver through a D/A converter (Edirol UA-25, Roland, Japan) controlled by Adobe Audition 3.0 (Adobe Systems Inc., Mountain View, CA, USA) on a laptop. The hydrophone was connected both to a preamplifier (Yamaha MLA8, Yamaha Music France, Marne-la-Vallée, France) linked to a video capture card of a PC (Osprey-450e) and to the laptop via the Edirol UA25 A/D converter (16 bit, 8 kHz) controlled with Adobe Audition 3.0. The video capture card synchronized the audio with the video signals obtained from a video camera (BUL520, brand, Active Media Concept, Vallauris, France) positioned in front of the setup. The video captured both the subject and opponent behaviours (Movie 1).

### Playback protocol

Unfamiliar males matched in size (i.e. SL ratio≤5%) were placed one in each aquarium c. 24 h before the experimental trials to allow them to set up territories. During this period, fish were visually and acoustically isolated from each other. Five minutes before the beginning of a trial, electrical appliances (except light) were switched off to reduce background noise. A trial began by removing the opaque partition allowing visual contact between males. The experimental protocol consisted in three successive periods of 5 min each: pre-playback (PRE), acoustic stimuli presentation (PBK) and post-playback with no acoustic stimuli presentation (POST). PRE is a control in which only visual stimuli are presented and POST tests for carry-over acoustic playback effects. During the PBK period, 10 acoustic stimuli were played back interactively, i.e. a sound was delivered when the opponent approached the subject and performed a lateral display or quivered (Movie 2); these behaviours typically accompany the production of agonistic sounds (Simões et al., 2008). This protocol thus attempted to simulate a natural agonistic sound production by the opponent (Movie 1). Note that sounds emitted by the test male or the underwater speaker could not be heard by the opponent in its aquarium and vice-versa (see below for measurements of amplitude levels and Fig. 3). Each subject fish was tested only once. After the trial the subject was removed, the opponent was moved to the subject's aquarium, and a new unfamiliar male was placed in the opponent's tank.

### Experiment 1 – frequency manipulation

We tested whether male fish use size information available in the spectral content of agonistic sounds by playing back acoustic signals of smaller and larger males (Fig. 4), while allowing visual access to a size-matched opponent. Subject male SL ranged from 8.4 to 9.5 cm (mean=9.0 cm; *n*=42 individuals). As playback stimuli, we used two agonistic sounds of the same male per trial (five renditions of each sound per trial delivered in a random order). These were randomly assigned either from four large [mean (range) SL=10.5 (10.2–10.8) cm] or from four small [SL=6.4 (6.2–6.6) cm] males from the ENES sound archive (Bertucci et al., 2012). The relative size difference of the eight males in the sound archive was paralleled by differences in their sounds' instantaneous frequencies; LF=291 (257–325) Hz and HF=606 (540–638) Hz (Bertucci et al., 2012). Frequency range (measured 3 dB below peak, FFT 1024 pts, Hanning Window) ranged from 334–759 Hz in HF and 231–371 Hz in LF signals. Each agonistic sound included seven to nine pulses (mean=8.0 pulses). We additionally used WN and silence (SIL) as controls. WN stimuli were 110 ms continuous sounds with 5 ms rise and fall times. WN did not contain pulse pattern amplitude modulation but had similar acoustic energy [average root mean square (RMS) amplitude] to agonistic sounds. All agonistic sound stimuli were equalized for peak amplitude. The amplitude of played-back sounds was adjusted to mimic a similar-sized male, producing agonistic sounds at a distance of approximately 4 cm, i.e. half the distance between the hydrophone and the front glass. This was a typical distance observed in subjects when interacting with the opponents. Each subject male received a single treatment (HF, LF, WN or SIL) and sounds from only one male or one WN file. The different acoustic stimuli were presented pseudo-randomly to the different subjects.

### Experiment 2 – amplitude modulation

This experiment was similar to experiment 1, except that we tested whether sound amplitude, a feature typically related with body size (Ladich, 1998; Amorim et al., 2013; also see above data for three males), was used in male–male assessment.

Playback experiments tested the effects of sound stimuli delivered at 6 dB above or below stimuli in experiment 1. Note that we cannot conclude that sound amplitude changes by c. 12 dB per 1 cm of SL since we only measured sound level in three individuals (see above). However, we used a fourfold change in amplitude, likely representing sounds from smaller and larger fish. We used two agonistic sounds per male from three males (with 8.4 cm, 9.0 cm and 9.3 cm SL) recorded during experiment 1 with eight (±1) pulses. We chose to use sounds from these males as they were size-matched with subject fish. Four WN stimuli were used as controls. Stimuli were equalized for peak amplitude at either HA or LA, and played back pseudo-randomly, except for WN, which was only delivered at HA. Subject male SL ranged from 7.9 to 9.2 cm (mean=8.6 cm; *n*=31 individuals).

**Fig. 5.**
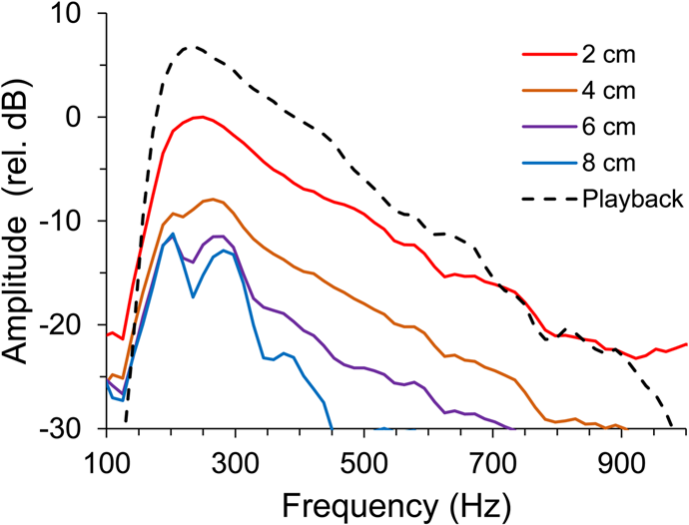
**Attenuation of agonistic fish sounds (LF) with distance along the speaker axis (zz in Fig. 3).** Two centimetres corresponds to the closest position of the hydrophone to the speaker. The frequency spectrum of the original sound file used for playback is represented for comparison. Note that, in contrast with sound amplitude, the frequency spectrum remained relatively invariant with distance. Spectra were calculated with a 4096 points FFT, Hanning window.

As sound amplitude decreases rapidly away from the source, we mapped amplitude attenuation in the interaction area using the aforementioned sound playback and recording chain (Fig. 3A). We measured average RMS power amplitude from sound recordings with a built-in function from Adobe Audition 3.0 at 2, 4, 6 and 8 cm in front of the hydrophone (z axis, Fig. 3B) and also around the typical location of the subject fish, i.e. in the middle of the interaction area, 4 cm in front of the hydrophone. Sound amplitude was measured above this middle point at 4 and 8 cm and below at 4 and 6 cm (y axis, Fig. 3C), and also to the left at 4 cm, and to the right at 4 and 8 cm (x axis Fig. 3D). For these measurements, two sound stimuli each from two males (with 8.4 cm and 9.0 cm SL) were used for playback. Sound amplitude attenuated mostly in the speaker axis (z) by 10 dB at 8 to 10 cm (at the front glass). In contrast, along the x-axis, amplitude increased by 2 dB near the opponent glass but attenuated rapidly in the opposite direction. Along the vertical y-axis, sound amplitude decreased by 3 dB near the substrate but remained practically constant in the first 4 cm above the reference point, decreasing up to 5 dB at 8 cm towards the surface. In contrast with sound amplitude, the frequency spectrum remained relatively invariant with distance (Fig. 5).

### Behavioural analysis

We analyzed videos using EthoLog (v2.2; Ottoni, 2000). We tallied total agonistic behaviour as the sum of sound, lateral display, quiver and bite events, from both the subject and the opponent observed during each 5-min trial period (for a full description of behaviours, see Simões et al., 2008; see also Movies 1 and 2). To ensure that we only used territorial subject males in the analysis, we only considered subjects that made more than nine agonistic behaviours, which was the first quartile of the observed behaviour for all subject males during the PRE period in experiment 1. With this criterion, we considered 31 fish in experiment 1 and 29 in experiment 2.

### Statistical analysis

To test the effect of playback treatment on total agonistic behaviour of subject males, we used linear mixed models with playback treatment, trial period, the interaction between treatment and trial period, and total agonistic behaviour of the opponent males for each experimental period as fixed effects, and subject as a random effect. We used the restricted maximum likelihood approach to fit the models. Treatment was included in the model as a factor and trial period and opponent agonistic behaviour as covariates. The latter was included to remove the effect of the opponent's behaviour on the subject. We included both random intercept and random slope parameters in the models (using an unstructured covariance matrix), i.e. we considered random intercept+slope models. However, the random slope parameters were not significant (experiment 1: Wald Z=0.88, *P*>0.05; experiment 2: Wald Z=1.66, *P*>0.05); only the random intercept presented a significant effect (experiment 1: Wald Z=2.32, *P*=0.02; experiment 2: Wald Z=3.11, *P*=0.002). Furthermore, the model for experiment 1 data did not converge. We therefore repeated the models including only the random intercept and using an identity covariance matrix structure. The random intercept+slope models and the random intercept models presented similar results for both experiments.

In the above analyses, we were especially interested in the interaction between treatment and trial period, which indicates if different levels of playback treatment modulate agonistic interactions differently. Therefore, when the LMM model was significant (in experiment 1) we then compared the slope for each treatment, i.e. the interaction term for each treatment (e.g. low frequency×trial period) with the baseline slope (silence×trial period in experiment 1). Model-fit was verified by visual inspection of the residual-plots. All tests were done with IBM SPSS 25.

### Ethical note

All experiments were performed in accordance with relevant guidelines and regulations including French national guidelines, permits and regulations regarding animal care and experimental use (approval no. D42-218-0901, ENES lab agreement, Direction Départementale de la Protection des Populations, Préfecture du Rhône).

## Supplementary Material

Supplementary information
