# Supplementary material for: Assessment of fighting ability in the vocal cichlid Metriaclima zebra in face of incongruent audiovisual information
Source: Biol Open. 2019 Dec 18;8(12):bio043356. doi: 10.1242/bio.043356 (PMC6955207; doi:10.1242/bio.043356)
Supplement: Supplementary information [file biolopen-8-043356-s1.pdf]

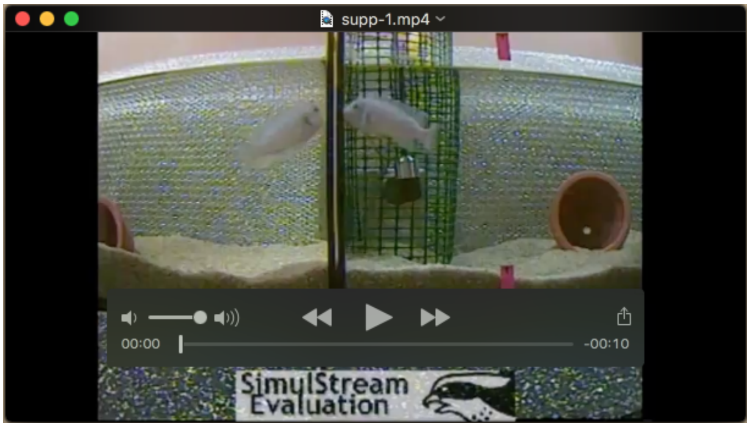

Movie 1. The movie shows two males interacting in the experimental set-up where the subject male (on the right) produces a sound during a lateral display.

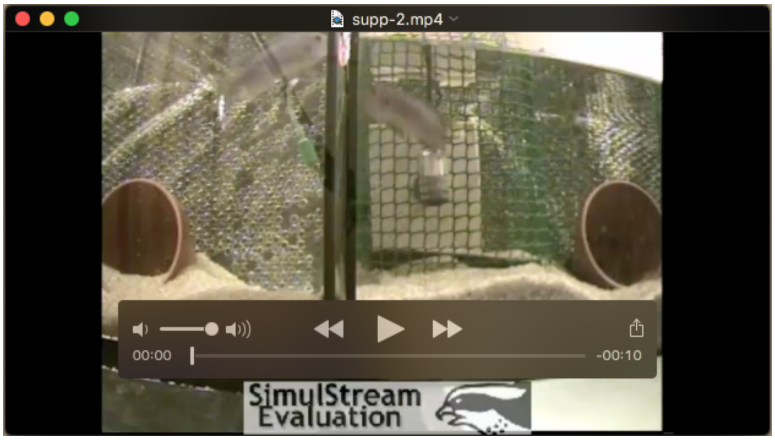

Movie 2. The movie depicts two males interacting during the playback period. An agonistic sound stimulus is rendered when the subject male (on the right) makes a lateral display.

Table S1. Experiment 1 and experiment 2 raw data.
Data concern subject fish size, and total agonistic behaviour of the subject fish and the opponent during the three periods of the experiment. For more details see Materials and Methods.

Click here to Download Table S1

Biology Open • Supplementary information
